# 933. Comprehensive Positioning and Splinting for the Burn Critical Care Nurse: Restructuring Interdisciplinary Training Following Turnover

**DOI:** 10.1093/jbcr/irag033.500

**Published:** 2026-04-06

**Authors:** Deborah Knight, Leslie W Poovey, Shakora Collins

**Affiliations:** Joseph M. Still Burn Center at Doctors Hospital, Augusta, GA; Joseph M. Still Burn Center at Doctors Hospital of Augusta / HCA Healthcare Center for Clinical Advancement, Augusta, GA; Doctors Hospital of Augusta Georgia, Augusta, GA

## Abstract

**Introduction:**

In 2021 the Burn Rehabilitation team at our ABA Verified Burn Center collaborated with the Burn Nursing leadership to develop competency training for 100 Burn ICU nurses on their role in the Burn Survivor’s rehab plan of care. At the time, there was a 99% pass rate of these competencies, with a noted decrease in pressure injuries within the unit. However, between January 2024 and April 2025, we identified 4 nerve compression injuries and 13 pressure wounds directly related to prolonged poor positioning or improper application of custom fabricated splints. In the three years since the initial competency training, our facility has only retained 20 of the Burn ICU nursing staff who attended the course, with 55 new nurses as of May 2025.

**Methods:**

Assessment of the original course revealed gaps in foundational principles including the timeline of burn wound healing and scar formation, the impact of prolonged edema, the role of exercise in critical care, and the chronic condition of burn injuries. The course was increased to four hours, with focused teaching on foundations and increased time for hands-on learning. The curriculum was written by a Certified Burn Therapist, who also provided all the lectures. Three experienced bedside therapists were included in the hands-on lab sessions. The course was required for all nurses who had not yet completed one year in the Burn ICU or Step-Down Unit, and attendance was optional for all other Burn Nurses.

**Results:**

5 four-hour sessions were provided to 36 nurses (37% of BICU and Step-Down nurses), 4 nurse leaders, and 1 Child Life Specialist. Only 30 participants (73%) responded to the course feedback form. 60% of respondents reported that this was their first nursing job, and the average number of years worked in Burn Critical Care was 4.5 (median 1 year) with experience ranging from 2 weeks to 25 years. The course received a satisfaction rating of 4.93 on a scale of 5. The Positioning module was rated the highest in perceived value (77% ranked 1st or 2nd), but when asked to share a key takeaway, the majority of responses (59%) were related to splinting or edema management. When asked to share one way they wished to change their practice, additional themes emerged including teamwork, early mobility, and mentorship.

**Conclusions:**

High nursing turnover rates in the Burn Critical Care setting leads to a gap in foundational knowledge and essential Burn Nursing Competency. By assessing the root cause of injuries acquired during the Critical Care stay in the context of turnover and new hire onboarding, customized curriculum can be modified to improve quality outcomes for Burn Survivors and lead to a reduction in hospital acquired injuries.

**Applicability of Research to Practice:**

Quality Improvement practices require ongoing assessment and modification in order to ensure best patient outcomes. Training on nursing's role in the patient's rehabilitation plan of care must be included in onboarding.

**Funding for the study:**

N/A.

